# Proportionate Incidence of Typhoid and Paratyphoid as Influenced by Vaccination

**Published:** 1916-11

**Authors:** 


**Proportionate Incidence of Typhoid and Paratyphoid as Influenced by Vaccination.** Marcel L'Abbe, Ann. de Med. Jan.-Feb. 1916, gives the following estimates, although conceding that it is based on reports not always absolutely determined: In the unvaccinated, 87.8% of the typhoid group are genuine typhoid, 10.9% paratyphoid B, 1.1% paratyphoid A. After 1 injection the cases are equally divided between true typhoid and paratyphoid B; after 2 injections, only 9.5% are true typhoid; after 3 injections 8.5%; after 4 injections true typhoid occurs in only 4.7%, paratyphoid B in 92.9% and paratyphoid A, too rare to be estimated in the preceding, 2.4%. While, after vaccination, the initial symptoms may be severe, the course of the disease is short and the later symptoms mild. (Note: Anti-vaccinationists have used the clinical reports of the present war as an argument against this method. There is no question but that enthusiasts have claimed too much for anti-typhoid inoculation, basing their claims on experience in peace under conditions when typhoid would not be expected at all or to a very insignificant degree at most. The charge has also been made of attempts to cover up with another diagnosis, cases of typhoid occurring after vaccination. The war experience, not yet reduced to accurate statistics of large numbers, unquestionably shows that anti-typhoid inoculation is not an absolute prophylactic, although failure has been shown to be due in considerable measure to incomplete protection, as by one or two injections of vaccine, or even to errors in histories, stating that vaccination had been performed when as a matter of fact, it had been omitted. But, practically all clinical experience in the war zone attests the great value of inoculation). It should be borne in mind that the present war is unique as regards continuous trench occupation, with little change from month to month, lack of opportunity for burying corpses—and difficulty of keeping them buried—conveyance of infectious material by rats and flies, and hardships practically preventing the carrying out of measures to insure sterility of water, milk and food. Without vaccination, under the present war conditions, even with full knowledge of sanitary methods of prophylaxis and competent executive efforts at control, it can scarcely be doubted that the typhoid incidence would have equaled that of our Spanish war in concentration camps. This latter has been cynically stated to amount to the infection of every man not immune from a previous attack and, for many regiments, even this statement was hardly an exaggeration).

